# System integration of magnetic medical microrobots: from design to control

**DOI:** 10.3389/frobt.2023.1330960

**Published:** 2023-12-19

**Authors:** Junjian Zhou, Mengyue Li, Na Li, Yuting Zhou, Jingyi Wang, Niandong Jiao

**Affiliations:** ^1^ State Key Laboratory of Robotics, Shenyang Institute of Automation, Chinese Academy of Sciences, Shenyang, China; ^2^ Institutes for Robotics and Intelligent Manufacturing, Chinese Academy of Sciences, Shenyang, China; ^3^ University of Chinese Academy of Sciences, Beijing, China; ^4^ College of Information and Electrical Engineering, Shenyang Agricultural University, Shenyang, China

**Keywords:** magnetic actuation, medical microrobot, control system, design system, system integration

## Abstract

Magnetic microrobots are ideal for medical applications owing to their deep tissue penetration, precise control, and flexible movement. After decades of development, various magnetic microrobots have been used to achieve medical functions such as targeted delivery, cell manipulation, and minimally invasive surgery. This review introduces the research status and latest progress in the design and control systems of magnetic medical microrobots from a system integration perspective and summarizes the advantages and limitations of the research to provide a reference for developers. Finally, the future development direction of magnetic medical microrobot design and control systems are discussed.

## 1 Introduction

Since the concept of medical microrobots was first proposed in Feynman’s lecture in 1959, research on microrobots has increased. In the research, it was discovered that magnetic microrobots offer superior benefits for medical applications, such as penetration of magnetic fields, and harmlessness to the body, and can remotely control microrobots. Several studies have investigated the properties and selection of materials (Soto et al., 2022), others have focused on developing structures and manufacturing methods (Rajabasadi et al., 2021; Wang Z. B. et al., 2022d; Wang Z. H. et al., 2022e), and others aspire to study motion control (Yang and Li, 2020; Wang X. et al., 2022c; Jiang et al., 2022). Most of the studies focus on one part of the magnetic medical microrobots, but there are few review articles on integrating the entire system, including how to design magnetic medical microbots with specific properties and how to precisely control them to accomplish a specific medical task.

The design and control systems of magnetic medical microrobots are interrelated. Therefore, we must combine the two parts from a system integration perspective. This review focuses on the elements of magnetic medical microrobot design and control system integration to provide a reference for researchers to design magnetic medical microrobots for successful application *in vivo*.

In Section 2 the materials are classified, the functions and characteristics of the various materials are discussed, and the three main structures of magnetic medical microrobots (microhelicals, microspheres, and microscaffolds), as well as their manufacturing technology, are introduced. In Section 3 we first classify and summarize the magnetic actuation modules and then introduce mainstream imaging methods. Next, we divide the control methods into three types: open-loop, closed-loop, and autonomous controls, and discuss the advantages and disadvantages of the three control methods. Finally, in the conclusion and outlook section, future directions for the development of magnetic medical microrobots are proposed.

## 2 Design system of magnetic medical microrobots

The first step in the design process of magnetic medical microrobots is selecting the mechanism of movement, material, structure, and manufacturing method. Currently, research on the mechanism of movement is abundant (Dreyfus et al., 2005; Field et al., 2019; Zhou et al., 2021; Wang Z. B. et al., 2022d), therefore, it will not be introduced in this review. This section introduces the latest progress in materials, the three structures, and manufacturing methods.

### 2.1 Material

According to their usefulness, the materials that constitute a magnetic microrobot, are mainly divided into three categories: functional materials, scaffold materials, and materials that not only serve as scaffolds but also have the required functions (Zhu et al., 2022).

Functional materials are materials that can respond to external field stimuli. Magnetic materials are representative of functional materials. Magnetic materials are divided into hard and soft magnetic materials. Hard magnetic materials with their high remanence and coercivity, offer efficient propulsion for microrobots in weak magnetic fields. However, most hard magnetic materials have poor biocompatibility, such as Ni/Co/NdFeB (Bernasconi et al., 2018; Li et al., 2018; Goudu et al., 2020). A few hard magnetic materials have good biocompatibility, such as FePt (Bozuyuk et al., 2022; Dogan et al., 2022). Soft magnetic materials, such as Fe and superparamagnetic iron oxide nanoparticles (SPIONs) (i.e., Fe_3_O_4_, γ-Fe_2_O_3_) (Park et al., 2019; Noh et al., 2022), often have good biocompatibility and biodegradability. However, they cannot provide an effective propulsion force in a weak magnetic field. Magnetic microrobots are often generated by the integration of magnetic materials, which are functional materials, into three-dimensional structures composed of scaffold materials. The integration methods include (a) a magnetic layer on the surface of microrobots (Mayorga-Martinez et al., 2022), (b) nanoparticles in the form of a scaffold (Giltinan et al., 2021), and (c) hybridization in polymers such as hydrogels.

Scaffold materials are materials that are used to create the main body (three-dimensional structure) of a magnetic medical microrobot. Biocompatibility and biodegradability are the first factors considered in selecting the scaffold material. SiO_2_ is often used to manufacture the main body of spherical microrobots (Bozuyuk et al., 2021). Photoresists and photosensitive resins, such as SU-8, are also used to manufacture microrobots with three-dimensional structures; however, they are difficult to degrade *in vivo*. The new generation of microrobots is manufactured with polymers, such as poly (lactic-co-glycolic acid) (PLGA) (Kim et al., 2018), pentaerythritol triacrylate (PETA) (Lee et al., 2023) and poly (ethylene glycol) diacrylate (PEGDA) (Chen et al., 2023). Cells and microbes can also be used as scaffold materials (Alapan et al., 2018).

The third category of materials is the intersection of functional and scaffolding materials. For example, (Alcântara et al., 2019) fabricated fully iron magnetic microrobots, Iron is a magnetic material and constitutes the main body of this magnetic microrobot, which means that the magnetic material that serves as a scaffold material belongs to the third class of materials. The smart hydrogel also belongs to the third material. While serving as the body of a magnetic microrobot, it can also change shape or volume in response to external field stimuli to accomplish complex medical tasks (Fusco et al., 2015; Lee et al., 2021; Xin et al., 2021). Chitosan (CHI) (Maria-Hormigos et al., 2023) and poly (N-isopropyl acrylamide) (PNIPAM), are the main representatives.

We customized the magnetic microrobots by selecting materials that correspond to the desired performance requirements. For example, if a magnetic material is integrated into a three-dimensional structure fabricated from a smart hydrogel, we will obtain a magnetic soft microrobot with deformation capabilities.

### 2.2 Structure and manufacturing method

**FIGURE 1:**
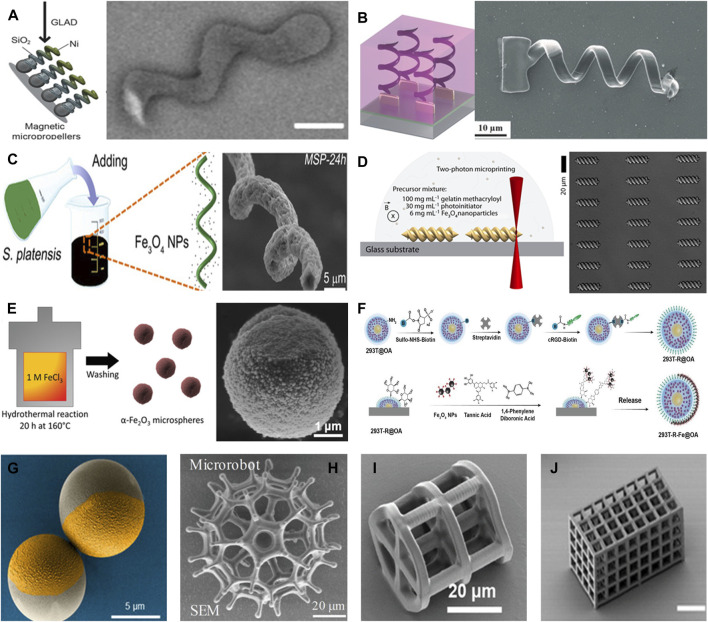
Structures and manufacturing technology. **(A)** GLAD technology. Adapted from Walker et al. (2015). Copyright 2015 The Authors under the CC-BY 4.0 license. **(B)** Template-assisted electroless deposition technology. Adapted from Zeeshan et al. (2013). Copyright 2013 John Wiley and Sons, Inc. **(C)** Bio-templated method technology. Adapted from Yan et al. (2017). Copyright 2017 AAAS. **(D)** DLW technology. Adapted from Ceylan et al. (2019). Copyright 2019 The Authors under the CC-BY 4.0 license. **(E)** α-Fe2O3-based microspheres. Adapted from Urso et al. (2021). Copyright 2021 John Wiley and Sons, Inc. **(F)** Cell-based microsphere. Adapted from Cong et al. (2022). Copyright 2022 John Wiley and Sons, Inc. **(G)** SiO2-based microspheres. Reprinted from Alapan et al. (2020). Copyright 2020 AAAS. **(H)** Porous spherical microrobot. Reprinted from Wei et al. (2020). Copyright 2020 John Wiley and Sons, Inc. **(I)** Porous cylindrical microrobot. Reprinted from Gyak et al. (2019). Copyright 2019 John Wiley and Sons, Inc. **(J)** Porous rectangular microrobot. Reprinted from Jeon et al. (2019). Copyright 2019 AAAS.

The manufacturing technology for microhelical structures is mature, and includes glancing angle deposition (GLAD) (Walker et al., 2015), template-assisted electroless deposition (TAED) (Zeeshan et al., 2013; Bernasconi et al., 2022), bio-templated methods (Yan et al., 2017), and direct laser writing (DLW) (Ceylan et al., 2019) (Figures 1A–D). Microhelical microrobots are propelled by rotating magnetic fields and can achieve three-dimensional motion in liquid environments, and (Xu et al., 2022) ensure the accuracy of 3D motion by compensating for angles.

Because of their excellent motion ability and simple dynamics model (Wu et al., 2022), microsphere microrobots have been used by many researchers in the study of motion control and targeted delivery of microrobots. The motion model of the microsphere is similar to that of the vehicle, and the motion control method of the vehicle can also be applied to the microsphere. Researchers commonly use iron oxide (Urso et al., 2021), living cells (Cong et al., 2022), and SiO_2_ (Alapan et al., 2020) (Figures 1E–G) as the main bodies of microspheres. Microspheres can often be manufactured by hydrothermal reactions, emulsion methods, and drop casting methods, and magnetic materials are subsequently attached to the surface of the microsphere by magnetron sputtering to generate propulsion capability.

The structure of a microscaffold is complex; therefore, it is typically manufactured using DLW technology. (Wei et al., 2020). used DLW technology to create a porous spherical microrobot that could transport engineered stem cells to the desired location for targeted therapy (Figure 1H). Similarly, cylindrical (Gyak et al., 2019), rectangular (Jeon et al., 2019) (Figures 1I, J), and other porous scaffold structures have been manufactured.

Microhelices and microscaffolds are often used for targeted drug/cell transport owing to their strong loading capacities (Dong et al., 2020). However, the structure of the two is complex, and it is difficult to further reduce its size. The advantages of microspheres are that they are simple to manufacture and can be submicron in size to reach complex, narrow areas of deep tissue. Simultaneously, because the movement area is mainly the blood vessel wall, it is less affected by blood flow and thus has a strong movement ability.

In addition, there are numerous shapes and structures, such as half-moons (Liu et al., 2023), dumbbell shapes, and deformable structures. Researchers can select materials, structures, and manufacturing methods according to the needed performance, driving magnetic field, moving environment, and other aspects for manufacturing magnetic medical microrobots.

## 3 Control system of magnetic medical microrobots

After preparing magnetic medical microrobots, precise control of microrobots to complete various medical tasks has become a focus of attention. In this section, we divide the control system of the magnetic medical microrobot into three parts: magnetic actuation, imaging, and control modules, and we summarize and discuss the research progress of each module.

### 3.1 Magnetic actuation module

A magnetic actuation module that can output uniformly variable magnetic fields is the premise of accurately controlling the motion of the microrobot. Presently, the mainstream magnetic actuation modules are permanent magnet and electromagnetic systems.

**FIGURE 2:**
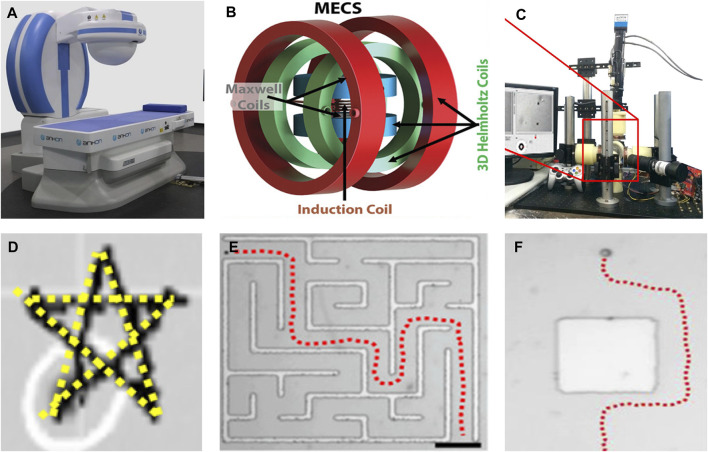
Magnetic actuation modules and control methods. **(A)** Single permanent magnet system. Reprinted from Liao et al. (2016). Copyright 2016 The Authors under the CC-BY 4.0 license. **(B)** Triaxial helmholtz coil. Reprinted from Ramos-Sebastian et al. (2022). Copyright 2022 The Authors under the CC-BY 4.0 license. **(C)** Five-coil electromagnetic system. Adapted from Wang et al. (2016). Copyright 2016 The Authors under the CC-BY 4.0 license. **(D)** PID control. Adapted from Tang et al. (2022). Copyright 2022 The Authors under the CC-BY 4.0 license. **(E)** The path planning algorithm Dijkstra. **(F)** Planning of obstacle avoidance paths. **(E,F)** reprinted from Li et al. (2017). Copyright 2017 American Chemical Society.

The magnetic field generated by the permanent magnet system is stable and the price is low. Various permanent magnet systems have been designed over the years, such as the single permanent magnet system (Liao et al., 2016) (Figure 2A), permanent magnet systems that can generate a rotating magnetic field(Mahoney and Abbott, 2014), and multi-permanent magnet systems (Ryan and Diller, 2017; Ginoya et al., 2021). However, the magnetic field strength of a permanent magnet is proportional to its volume. This makes the drive system bulkier, leading to increased costs and reduced flexibility. Moreover, the magnetic field of a permanent magnet system cannot disappear immediately, which may lead to certain safety risks in clinical operations.

Electromagnetic systems generate the needed magnetic field by setting the input current value and waveform. Paired coils are a mainstream electromagnetic system, consisting of Helmholtz coils and Maxwell coils (Ramos-Sebastian et al., 2022) (Figure 2B), similar jobs include (Wang et al., 2022b; Xu et al., 2023). The disadvantage of paired coils is that the ratio of the coil diameter to the distance between the coils is unchanged. Increasing the coil diameter to expand the working space results in a larger device size, compromising energy efficiency and weakening the magnetic field strength. Distributed electromagnetic systems are another type of mainstream electromagnetic system. It installs cylindrical coils symmetrically around the workspace and inserts an iron core inside the coil to enhance the magnetic field. The number of coils in a distributed electromagnetic coil system is typically five (Chen et al., 2022), six (Wang et al., 2016) (Figure 2C), and eight (Kummer et al., 2010; Le et al., 2016). The more the number of coils, the more degrees of freedom the microrobot has (the number of coils is less than 8). When the number of coils reaches eight, all six degrees of freedom of motion of the magnetic microrobot are controllable. The disadvantage is that the working space of the electromagnetic system is certain and cannot be moved. However, (Yang Z. X. et al., 2020b; Du et al., 2022; Zhang et al., 2023), combined an electromagnetic system with a robotic arm to increase the working space. When an electromagnetic system works for a long time, it will produce a high temperature and thus may damage the system equipment. Therefore, the electromagnetic system usually needs to be equipped with an additional cooling system.

A permanent magnet system has a large working space; therefore, it is suitable for controlling magnetic medical microrobots with a wide range of motion. However, magnetic microrobots move with low precision and few degrees of freedom in a permanent magnet system. The electromagnetic system has numerous degrees of freedom; therefore, it can precisely control the robot to complete various complex movements; however, because the working space is fixed, the application is limited to the operation of local areas.

### 3.2 Imaging module

At present, most research experiments involve *in vitro* environments, and CCD cameras can be used to observe the size of microrobots at the millimeter and submillimeter levels (Xu et al., 2020). Microscopes and cameras are often combined to observe microrobots at micron or nanometer scales (Li et al., 2020).

When the motion scene of the microrobots is in the body, the camera cannot observe the position and state of the microrobots. Therefore, we need to adopt other imaging positioning technologies to locate the robot.

If the movement range of the microrobot is in shallow tissue, such as subcutaneous tissue, the optimal choice is fluorescent imaging (FI) (Yan et al., 2017) technology and ultrasonic (US) imaging technology (Wu et al., 2018; Wang et al., 2022a). The tissue penetration ability of the two technologies is limited; however, they are cost-effective and offer excellent real-time performance.

When a microrobot is in deep tissues, magnetic resonance imaging (MRI) technology with excellent tissue penetration ability is an excellent choice. (Tiryaki and Sitti, 2022). used MRI technology to locate the position of the microrobot in real-time in an *in vitro* pig kidney. MRI can also be used for postoperative imaging (Go et al., 2022). X-ray technology is also an excellent choice. (Nguyen et al., 2021). designed a microrobot loaded with doxorubicin (DOX) to autonomously navigate to the pathological position under X-ray imaging to complete drug release. However, the drawback of X-rays is that they produce strong ionizing radiation. Imaging cannot be performed over an extended period or in real time.

In addition to the imaging methods discussed above, imaging methods include magnetic (Son et al., 2016; Xu et al., 2021; Le et al., 2023) and radio frequency positioning methods (Bao et al., 2015; Hoang et al., 2023). The spatial position information of the microrobot was obtained by collecting and processing magnetic field information/high-frequency electromagnetic waves. However, the disadvantage of this method is that the relative position information between the microrobots and the organization cannot be obtained.

### 3.3 Control module

We divide the control methods into three categories (open-loop, closed-loop, and autonomous controls). The first type of control was open control. Open-loop control means that the operator manually controls the motion of microrobots. (Jeon et al., 2019).used an open-loop method to control the movement of porous microrobots loaded with stem cells in a mouse cerebral vascular model. Although the open-loop control mode is simple and convenient to operate, the error is large, and it is difficult to control the movement accurately.

The second type of control is closed-loop control. Currently, various control algorithms have been applied to magnetic medical microrobots, such as proportional integral derivative (PID) control (Tang et al., 2022) (Figure 2D), robust control, model predictive control (MPC) (Yang et al., 2021), sliding mode control (Mousavi et al., 2022), and backstepping control (Fruchard, 2023). The operator still must help the microrobot to identify the target and plan the trajectory, therefore, its control is not completely automatic and intelligent, and we also call the closed-loop control semi-autonomous control.

The third type of control is autonomous control. Autonomous control has a planning and decision-making component in addition to the sensing and control component, which enables magnetic microrobots to have autonomous trajectory planning, self-learning, and self-decision-making capabilities. It is difficult for microrobots that move only along a fixed trajectory to complete complex medical tasks; thus, an autonomous control method is needed for the next-generation of microrobots.

We often use global path planning algorithms to determine a collision-free optimal path for a magnetic microrobot to ensure that it can reach the target area quickly, as (Liu et al., 2020) used the informed optimal random exploring tree (Informed RRT*) algorithm to provide efficient and reliable obstacle avoidance paths for spiral microrobots. In subsequent work (Liu et al., 2021), the RRT*−Connect algorithm was used to plan a smooth and shortest path in space, similar to A-star (A^*^) (Fan et al., 2018) and PSO (Yang L. D. et al., 2020a). However, the global path planning algorithm is only applicable in static environments and cannot make decisions regarding dynamic obstacles. In complex and dynamic *in vivo* environments, hybrid global and local path planning is an optimal choice, (Li et al., 2017), used the Dijkstra algorithm to achieve global path planning (Figure 2E), while using analog logic methods to change the trajectory of magnetic microrobots when encountering dynamic obstacles, thus achieving dynamic obstacle avoidance (Figure 2F). Machine learning and deep learning have also been integrated into magnetic medical robots, empowering them with self-learning and enhanced environmental adaptability (Behrens and Ruder, 2022; Yang et al., 2022).

Among these three control methods, closed-loop control is currently the most suitable control metho. Although autonomous control offers the robot the ability to plan and make decisions, it can make faster and more accurate plans than the human brain. However, it overly relies on algorithms and is not as stable as the human brain. Hence, we contend that human involvement in the control system ensures safety.

## 4 Conclusion and outlooks

This review introduces the design and control systems of magnetic medical microrobots and divides the materials composed of magnetic medical microrobots into three categories. Here, we provide examples of the members and functions of the three materials. Fabrication methods for the three main structures of magnetic medical microrobots are also introduced. Subsequently, we analyze and discuss the control system using three modules. This study provides a reference for developers to design and control magnetic medical microrobots. Table 1 summarizes the characteristics of each module of a magnetic medical microrobot. Based on the needed performance and medical functions, we can select suitable materials, structural fabrication techniques, magnetic drive modules, imaging modules, and control methods. This allows for the comprehensive design of the robot and the construction of its control system.

**TABLE 1:** Design and control system integration of magnetic medical microrobots.

		Common members		Advantage	Disadvantage	Applications
Design system	Material	Functional material	Magnetic material that is nanoparticles in the form of a scaffold	They can respond to external fields	Most magnetic materials are difficult to combine biocompatibility and propulsion capabilities Deformation degrees of freedom of smart hydrogels are less	Functional materials can respond to external fields to change their shape or volume, or to generate propulsion for magnetic microrobots
			Magnetic material that is a magnetic layer on the surface of microrobots			
			Magnetic material that is hybridization in polymers such as hydrogels			
		materials that not only serve as scaffolds but also have the required functions	Smart hydrogel	It plays multiple roles on magnetic medical microrobots	The deformation freedom of smart hydrogels is less	These materials can be used for magnetic microrobots fabricated from all-magnetic materials, and soft magnetic microrobots
			Magnetic material as support materials			
		Scaffold material	SiO2, Photoresists and photosensitive resins, Polymer, Cell and microbe	They can provide a structure that is difficult to damage	This requires better biocompatible and biodegradable materials	Scaffold materials as materials for the fabrication of three-dimensional structures for magnetic medical microrobots
	Common Structures	Microhelical	—	High propulsion capacity, large surface area, high load	—	Targeted Delivery, thrombus removal
		Microsphere		Simple motion modeling, easy to manufacture, fast motion speeds	—	Targeted Drug Delivery, single-cell operation
		Microscaffold		Large specific surface area, high-loading	—	Cell Culture and Delivery Targeted Drug Delivery
Control system	Magnetic actuation module	Electromagnetic system	Four/five/eight coil electromagnetic systems. Triaxial Helmholtz coil, etc.	Multiple control degrees of freedom, high control accuracy	Fixed working space, low energy conversion efficiency	Local Area Operation, high precision/intelligent motion of robots
		Permanent magnet system	Single-permanent Permanent Magnet Systems	Large working space, no heat generation, Low cost	Large magnetic field strength requires large permanent magnets, magnetic field cannot be turned off, complex motion control	Wide-area operation, rapid verification of robot kinematics
			Multi-permanent Magnet Systems			
	Common imaging module	Microscope/CCD	—	High resolution, good real-time	Impervious	Vitro environment
		US		Good real-time performance, low cost	Weak penetration, poor resolution	Subcutaneous tissue, isolated tissue, etc.
		FI		Highly visible imaging results, good real-time performance	Weak penetration	Subcutaneous tissue, mouse peritoneal membrane, and other superficial tissues
		MRI		High resolution, actuate, and imaging can be balanced	Poor real-time performance	Intracranial, hepatic, vascular
		X-ray/CT		high resolution	Radioactive and damaging to tissues	deep-rooted organization
	Control module	Open-loop control	—	Simple control	Low control accuracy	Scenes with open environments and large target areas
		Closed-loop control		High control accuracy, strong interaction capability	Inability to autonomously plan movement trajectories	Application scenarios requiring motion accuracy
		Autonomous control		Ability to self-learn exercise judgment and adapt to the environment	Poor interactivity, motion is algorithm-dependent and security needs to be improved	Application scenarios that require motion accuracy and complex and changing environments

Although microrobots have been tested *in vitro*, and several have been tested in animals, they are still not qualified for actual clinical application (Soto and Chrostowski, 2018). Future developments of magnetic medical microrobots include the following: 1) The materials of magnetic medical microrobots should meet the standards of practical biomedical applications and improve their biocompatibility and biodegradability. 2) Group magnetic medical microrobots can achieve various motion modes such as collaboration and modules, and perform more complex medical tasks, which is an important direction in the research and development of magnetic medical microrobots. (Wang et al., 2022b; Yu et al., 2022; Mayorga-Martinez et al., 2023). have been studying the motion of group magnetic medical microrobots. 3) Intelligent and multi-degree-of-freedom development of magnetic medical microrobots is necessary, with self-examination, learning ability, variability ability, and the ability to adapt to the changeable and complex environment by changing the shape or motion state. Through detailed research on the design and control systems of magnetic medical microrobots, we think that magnetic medical microrobots will become a daily presence, safeguarding our health.
